# Clinical features of IgG4-related periaortitis/periarteritis based on the analysis of 179 patients with IgG4-related disease: a case–control study

**DOI:** 10.1186/s13075-017-1432-8

**Published:** 2017-10-04

**Authors:** Makiko Ozawa, Yasunari Fujinaga, Junpei Asano, Akira Nakamura, Takayuki Watanabe, Tetsuya Ito, Takashi Muraki, Hideaki Hamano, Shigeyuki Kawa

**Affiliations:** 1 Department of Gastroenterology, Shinshu University School of Medicine, 3-1-1 Asahi, Matsumoto, 390-8621 Japan; 2 Department of Radiology, Shinshu University School of Medicine, 3-1-1 Asahi, Matsumoto, 390-8621 Japan; 3 Department of Internal Medicine, Matsumoto Dental University, 1780 Gobara, Hirooka, Shiojiri, Nagano 399-0781 Japan

**Keywords:** IgG4-related periaortitis/periarteritis, IgG4-related disease, Autoimmune pancreatitis, Activity marker

## Abstract

**Background:**

Immunoglobulin G4-related disease (IgG4-RD) is a newly recognized systemic condition characterized by high serum immunoglobulin G4 (IgG4) concentration and IgG4-bearing plasma cell infiltration in affected organs. Although it has become evident that IgG4-RD also involves the systemic aortic/arterial system, the precise details of this condition remain unclear. The present study sought to clarify the clinical features of IgG4-related periaortitis/periarteritis.

**Methods:**

Among 223 patients with IgG4-RD, 179 (131 male, median onset age 67 years) were recruited for this study. Periaortitis/periarteritis was defined as vessel wall thickness with circumferential enhancement on contrast-enhanced computed tomography.

**Results:**

Periaortitis/periarteritis was identified in 65 (36.3%; 53 male) of 179 IgG-RD patients. The distribution of IgG4-related periaortitis/periarteritis could be broadly classified into five types, with the most prevalent Type 2 (44.6%) being localized at the infra-renal artery portion of the abdominal aorta and continuing to the iliac arteries. The infra-renal artery region of the abdominal aorta was most frequently involved (>80%) among IgG4-related periaortitis/periarteritis cases. Comparisons of clinical parameters between IgG4-RD patients with and without periaortitis/periarteritis revealed significantly higher propensities for older IgG4-RD onset age and highly active disease state featuring elevated serum IgG, IgG4, circulating immune complex, and soluble interleukin-2 receptor. All patients showed improvement of wall thickening after steroid therapy, although nine patients (20.9%) exhibited worsening of luminal dilatation. The main risk factor for this manifestation was prior luminal dilatation according to multivariate analysis.

**Conclusion:**

IgG4-related periaortitis/periarteritis predominantly occurred at the infra-renal artery portion of the abdominal aorta, affected older IgG4-RD onset patients, and was prevalent in highly active disease states. As reported previously, the main risk factor for worsening luminal dilation after corticosteroid therapy was the existence of luminal dilation beforehand.

## Background

Immunoglobulin G4-related disease (IgG4-RD) is a systemic condition characterized by high serum immunoglobulin G4 (IgG4) concentration and IgG4-bearing plasma cell infiltration in affected organs [1–3] that is considered to be an immune-mediated disorder and possibly an autoimmune disease. The hallmark features of IgG4-RD can be summarized as systemic organ involvement, the ability to involve multiple organs either simultaneously or in a metachronous fashion, imaging findings of swelling, nodules, and/or increased organ wall thickness, elevated serum IgG4 concentration, lymphoplasmacytic and IgG4-bearing plasma cell infiltration within affected organs, and a generally favorable response to glucocorticoid therapy [1]. The concept of IgG4-RD was established through extensive evaluation of extra-pancreatic lesions complicating autoimmune pancreatitis (AIP) [3], a specific type of chronic pancreatitis and an original member of the IgG4-RD family. It is now widely believed that IgG4-RD characteristically manifests as Mikulicz’s disease [4], respiratory disorders [5], sclerosing cholangitis [6], retroperitoneal fibrosis [3], tubulointerstitial nephritis [7], and prostatitis [8]. Moreover, it has recently become evident that IgG4-RD also includes systemic cardiovascular disease, namely in the form of IgG4-related cardiovascular disease, which has been investigated using imaging modalities that include computed tomography (CT), magnetic resonance imaging (MRI), echocardiography, positron emission tomography using the glucose analog 2-deoxy-2-[^18^F] fluoro-d-glucose (^18^F-FDG-PET), and cardiac catheterization [9]. Intensive study of the aortic/arterial system has revealed another possible new disease concept, IgG4-related periaortitis/periarteritis.

Since 2008, several manifestations of IgG4-related periaortitis/periarteritis have been detected or recognized in case reports during systemic imaging analysis of other IgG4-RDs and pathological evaluation of surgical specimens, such as those for aneurysm. Consequently, a small number of chronic periaortitis cases involving the abdominal aorta have been reported in association with IgG4-RD [10–14]. Stone et al. [15] described two patients with ascending aortitis whose aortic histopathology was characterized by lymphoplasmacytic aortitis in which plasma cells stained intensely for IgG4. Ensuing systemic studies on IgG4-related arterial lesions have focused on pathological evaluation and clinical imaging analysis.

Regarding pathological evaluation, IgG4-RD was first detected in relation to inflammatory abdominal aortic aneurysm (AAA) [10, 11, 16, 17]. In contrast to lesions in the abdominal aorta, the thoracic aortic lesions of IgG4-RD were found to assume different forms with various clinicopathological features, including inflammatory aneurysm, lymphoplasmacytic aortitis, isolated aortitis, and aortic dissection [15, 18–20]. Furthermore, IgG4-related vascular lesions were observed to spread to medium-sized arteries, such as the coronary arteries, as well as to the first and second branching arteries of the aorta [12, 21, 22]. Histopathologically, IgG4-related arterial lesions exhibited arterial wall thickening corresponding to inflammation with IgG4-positive plasmacytes and fibrosis mainly in the adventitia [12, 17, 19, 21, 22].

The clinical evaluation of IgG4-RD has largely been carried out concomitantly with the systemic imaging analysis of other IgG4-RDs. Inoue et al. [21] reported periarterial lesions in patients with IgG4-RD involving the aorta or arteries that were predominantly located in the abdominal aorta to iliac arteries. Mizushima et al. [23] retrospectively evaluated the clinical features of IgG4-related aortitis/periaortitis and periarteritis on the basis of periaortic/periarterial radiological findings to propose organ-specific diagnostic and exclusion criteria. To some extent, these studies clarified the clinical aspects of IgG4-related arterial lesions, including their prevalence, symptoms, laboratory, imaging, and pathological findings, treatment, clinical course, and complications. It was also found that some patients with IgG4-related periaortitis/periarteritis having luminal dilatation of affected vessels before corticosteroid therapy experienced post-treatment exacerbation of this symptom [23].

In spite of the recent progress in defining IgG4-related periaortitis/periarteritis, extensive studies with sufficient case numbers are needed. In this context, the present study sought to clarify the clinical features of IgG4-related periaortitis/periarteritis as determined by the clinical and imaging analysis of 179 patients with IgG4-RD.

## Methods

### Patients

We enrolled 223 patients with IgG4-RD (165 male and 58 female, median age at IgG4-RD onset 66 years) who were seen at our clinic or affiliated hospitals between 2003 and 2015. IgG4-RD was diagnosed based on the Japanese Comprehensive Diagnostic Criteria for IgG4-RD [24] in all cases, among which 125 cases of AIP had been diagnosed based on the International Consensus Diagnostic Criteria 2011 [25] primarily due to characteristic imaging findings, high serum IgG4 concentration, the presence of extra-pancreatic lesions (i.e., other IgG4-RDs), and steroid responsiveness. Ultimately, 179 patients (131 male and 48 female, median age at IgG4-RD onset 67 years) were selected for further study based on the exclusion criteria of insufficient CT analysis, which included patients with impaired renal function who were contraindicated for the use of contrast medium for enhanced CT, prior corticosteroid therapy before admission, and age < 18 years.

### Methods

#### Detection of periaortitis/periarteritis by CT

All 179 patients underwent contrast-enhanced CT that was reviewed by an experienced radiologist (YF) at Shinshu University School of Medicine. Periaortitis/periarteritis was defined as vessel wall thickness and wall enhancement either partially of circumferentially on CT. We recorded maximum vessel wall thickness and lumen diameter in affected lesions. Improvement of periaortitis/periarteritis was defined as decreased vessel wall thickness. Luminal dilatation at the time of periaortic/periarterial lesion diagnosis was defined as that for aneurysm, in which the luminal diameter was more than 1.5 times wider than normal (i.e., > 45 mm at the thoracic aorta and > 30 mm at the abdominal aorta) according to the Japanese Circulation Society 2011 guidelines for diagnosis and treatment of aortic aneurysm and aortic dissection [26].

#### Risk factors for periaortitis/periarteritis in IgG4-RD

We analyzed for risk factors of periaortitis/periarteritis complications by comparing such clinical parameters as IgG4-RD onset age, gender, serum levels of various activity markers such as IgG4, IgG, complement proteins, soluble interleukin-2 receptor (sIL-2R), rheumatoid factor (RF), and circulating immune complex (CIC), number of lesions, and history of corticosteroid therapy between patient groups with and without periaortitis/periarteritis. Patients taking hypotensive agents were deemed to be hypertensive.

#### Outcome of corticosteroid treatment

We compared various clinical parameters between patients with and without exacerbation of luminal dilatation after corticosteroid therapy to assess the effects of treatment. The exacerbation of luminal dilatation was defined as an expansion of luminal diameter > 5 mm at the same site as periaortitis/periarteritis lesions detected at the time of the initial diagnosis [23].

### Statistics

Differences between groups were analyzed using the Mann–Whitney test for continuous data and the χ^2^ test or Fisher’s exact test for categorical data. Statistical analyses were performed using Stat Flex version 6 software (Artech Co., Ltd). *p* < 0.05 was considered statistically significant.

### Ethics

This study was approved by the ethics committee of Shinshu University School of Medicine (Approval Code: 3597). Because this study was a retrospective observational study with medical records, and was not an intervention study with human samples, consent to participate was not needed. Our ethics committee also approved that consent to participate was not needed. The investigation was conducted in compliance with the Helsinki Declaration.

## Results

### Prevalence of IgG4-related periaortitis/periarteritis

The study cohort included 179 patients with IgG4-RD (131 male and 48 female, median age at IgG4-RD onset 67 years). One hundred and nine patients received steroid therapy after the involved vital organs exhibited a risk of serious dysfunction or failure, such as obstructive jaundice due to pancreatic head swelling or urethral stenosis from retroperitoneal fibrosis, or when unbearable abdominal pain or other severe symptoms were present. Steroid treatment was carried out according to the Japanese Consensus Guidelines for AIP, which recommended a minimum of 3 years of maintenance therapy [27].

**Fig. 1:**
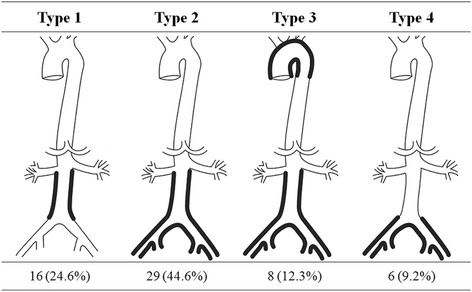
Classification of IgG4-related periaortitis/periarteritis distribution into five types. Type 1 localized at the infra-renal artery portion of the abdominal aorta (16 cases; 24.6%); Type 2 added continuation to medium-sized arteries, mainly the iliac arteries (29 cases; 44.6%); Type 3 added separate localization to the ascending aorta (eight cases; 12.3%); Type 4 affected medium-sized arteries only (six cases; 9.2%); and Type 5 was other involvement

Periaortitis/periarteritis was identified in 65 (36.3%; 53 male and 12 female) of 179 IgG4-RD patients and could be broadly classified into five types (Fig. 1, Table 1). Type 1 IgG4-related periaortitis/periarteritis was localized at the infra-renal artery portion of the abdominal aorta (16 cases; 24.6%), Type 2 added continuation to medium-sized arteries, mainly the iliac arteries (29 cases; 44.6%), Type 3 added separate localization at the ascending aorta (eight cases; 12.3%), Type 4 affected medium-sized arteries only (six cases; 9.2%), and Type 5 exhibited other involvement, including separate localization at the thoracic aorta, abdominal aorta, and medium-sized arteries (two cases), at the thoracic aorta and abdominal aorta (one case), at the abdominal aorta and medium-sized arteries (one case), at the aortic arch (one case), and continuing from the ascending aorta to the iliac arteries (one case). The most prevalent location was Type 2. Moreover, the infra-renal artery portion of the abdominal aorta was most frequently involved as seen in Types 1, 2, and 3, (> 80%), while the supra-renal artery portion of the abdominal aorta was largely spared.

**Table 1:** Distribution of IgG4-related periaortitis/periarteritis

Type	Distribution	Frequency
1	Localization at the infra-renal portion of the abdominal aorta	16 (24.6%)
2	As Type 1, with continuation to medium-sized arteries (mainly the common iliac arteries)	29 (44.6%)
3	As Type 2, with separate manifestation in the ascending aorta	8 (12.3%)
4	Localization in medium-sized arteries only	6 (9.2%)
5	Other localization pattern	6 (9.2%)
Total		65 (100%)

Periaortitis/periarteritis could be broadly classified into five types, and the most prevalent location was Type 2. Moreover, the infra-renal artery portion of the abdominal aorta was most frequently involved as seen in Types 1, 2, and 3, (> 80%), while the supra-renal artery portion of the abdominal aorta was largely spared

*IgG4* immunoglobulin G4

### Risk factors for periaortitis/periarteritis in IgG4-RD

To identify the risk factors associated with the onset of periaortitis/periarteritis, we compared several clinical parameters between IgG4-RD patients with and without periaortitis/periarteritis (Table 2). There were no significant differences in gender ratio between the groups, although the onset age of IgG4-RD was significantly higher in the positive group (69.1 vs 65.0 years, *p* = 0.00752). Laboratory tests revealed significant disease activity marker increases in the positive group for IgG, IgG4, CIC, and sIL2R. IgG remained significantly higher in the positive group (*p* = 0.0035, OR 1.0007, 95% CI 1.0002–1.001) according to multiple logistic analysis. Neither a history of smoking or hypertension nor levels of IgE, white blood cells (WBC), or C-reactive protein (CRP) showed a significant association with risk.

**Table 2:** Comparison of clinical findings between patients with and without IgG4-related periaortitis/periarteritis

	Univariate analysis			Multivariate analysis	
Parameter	Periaortitis/periarteritis (+)	Periaortitis/periarteritis (–)	*p* value	OR (95% CI)	*p* value
Total	65	114			
Onset age of IgG4-RD (median years)	69	65	0.0075	1.04 (1.00–1.08)	0.082
Sex (male/female)	53/12	78/36	0.057		
Median number of other organ involvements	3	3	0.63		
Allergy (+/–)	17/47	46/64	0.044	0.49 (0.23–1.05)	0.065
Smoking (+/–)	36/28	58/47	0.90		
Hypertension (+/–)	30/34	37/70	0.11		
Median IgG (mg/dl)	2266	1854	0.00015	1.0007 (1.0002–1.001)	0.0035
Median IgG4 (mg/dl)	511	376	0.012		
Median IgE (IU/ml)	172	100	0.12		
Median WBC (/μl)	6190	6090	0.87		
Median CRP (mg/dl)	0.17	0.13	0.68		
Median RF (U/ml)	7	6	0.73		
Median CIC (μg/ml)	5.3	4.8	0.042	0.96 (0.88–1.04)	0.29
Median sIL-2R (U/ml)	885	659	0.00049	1.0004 (1.00–1.001)	0.23

The onset age of IgG4-RD was significantly higher in the positive group. Laboratory tests revealed significant disease activity marker increases in the positive group for IgG, IgG4, CIC, and sIL2R. IgG remained significantly higher in the positive group according to multiple logistic analysis

*OR* odds ratio, *CI* confidence interval, *CIC* circulating immune complex, *CRP* C-reactive protein, *IgE* immunoglobulin E, *IgG* immunoglobulin G, *IgG4-RD* immunoglobulin G4-related disease, *RF* rheumatoid factor, *sIL-2R* soluble interleukin-2 receptor, *WBC* white blood cells

Regarding the complication of other organ involvement at IgG4-RD diagnosis, kidney and urinary tract involvement was significantly more frequently seen in the positive group in both univariate (*p* = 0.043) and multiple logistic analyses (*p* = 0.050, OR 2.06, 95% CI 1.00–4.24) (Table 3).

**Table 3:** Comparison of other organ involvement at diagnosis between patients with and without IgG4-related periaortitis/periarteritis

	Univariate analysis			Multivariate analysis	
Involved organ	Periaortitis/periarteritis (+)	Periaortitis/periarteritis (–)	*p* value	OR (95% CI)	*p* value
Total	65	114			
Lacrimal gland (+/–)	15/50	35/79	0.28	0.63 (0.9–1.37)	0.24
Salivary gland (+/–)	32/33	53/61	0.72	1.17 (0.59–2.35)	0.66
Respiratory organs (+/–)	26/39	40/74	0.51	1.36 (0.69–2.70)	0.37
Pancreas (+/–)	45/20	80/34	0.89	1.07 (0.51–2.28)	0.85
Biliary tract (+/–)	28/37	51/63	0.83	0.79 (0.39–1.59)	0.50
Kidney, urinary tract (+/–)	22/43	23/91	0.043	2.06 (1.00–4.24)	0.050
Prostate (+/–)	13/52	17/97	0.38	1.31 (0.56–3.06)	0.53
Lymph nodes (+/–)	41/24	75/39	0.71	0.80 (0.40–1.59)	0.52
Other organs (+/–)	11/54	16/98	0.60		

Regarding the complication of other organ involvement at IgG4-RD diagnosis, kidney and urinary tract involvement was significantly more frequently seen in the positive group in both univariate and multiple logistic analyses

*OR* odds ratio, *CI* confidence interval, *IgG4* immunoglobulin G4

### Outcome of corticosteroid treatment

**Fig. 2:**
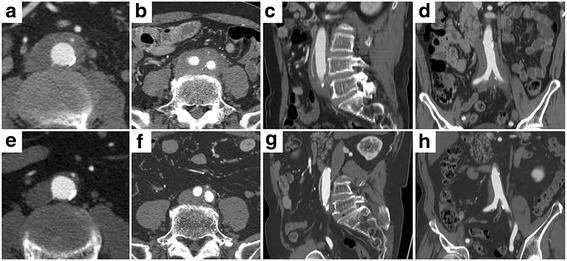
Contrast-enhanced CT (early phase) of IgG4-related perivascular lesions in an 80-year-old man. Walls of the abdominal artery (**a**) and common iliac arteries (**b**) showed thickening at diagnosis (**a, b** transverse plane; **c** sagittal plane; **d** coronal plane). Steroid therapy reduced abdominal wall thickness (**e, f** transverse plane; **g** sagittal plane; **h** coronal plane)

**Fig. 3:**
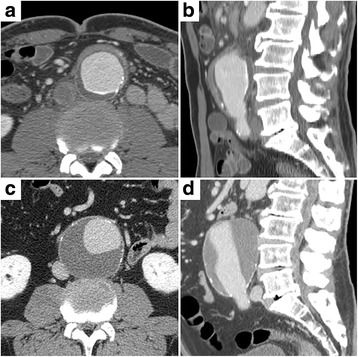
Contrast-enhanced CT (delayed phase) of IgG4-related perivascular lesion in a 72-year-old man. Walls of the abdominal artery showed thickening at diagnosis (**a** transverse plane; **b** sagittal plane). Steroid therapy reduced wall thickness but dilated the vascular lumen (**c** transverse plane; **d** sagittal plane)

Among the 65 IgG4-related periaortitis/periarteritis patients, 43 (66.2%) received corticosteroids. All patients showed improvement of wall thickening after therapy (Fig. 2), although nine patients (20.9%) also exhibited worsening of luminal dilatation (Fig. 3). Univariate analysis comparisons of the nine patients with luminal dilatation after steroid therapy and the 34 patients without revealed prior luminal dilatation before treatment to be associated with this symptom (*p* < 0.0001) (Table 4), which was supported by multiple logistic analysis (*p* = 0.0057, OR 93.0, 95% CI 3.7–2300). Among the nine patients with luminal dilatation, two underwent endovascular aortic repair and continued corticosteroid therapy without recurrence.

**Table 4:** Comparison between patients with and without IgG4-related periaortitis/periarteritis with exacerbation of luminal dilatation after therapy

	Univariate analysis			Multivariate analysis	
Parameter	Exacerbation (+)	Exacerbation (–)	*p* value	OR (95% CI)	*p* value
Total	9	34			
Median onset age of IgG4-RD (years)	67	69	0.26	0.83 (0.66–1.06)	0.13
Sex (male/female)	7/2	30/4	0.42	0.75 (0.0053–108)	0.91
Luminal dilatation before treatment (+/–)	6/3	2/32	< 0.0001	93.0 (3.7–2300)	0.0057
Allergy (+/–)	2/7	11/23	0.56		
Smoking (+/–)	7/2	21/13	0.37	6.4 (0.061–660)	0.43
Hypertension (+/–)	5/4	15/19	0.54	6.3 (0.44–90)	0.18
Median IgG (mg/dl)	2451	2903	0.86	1.0004 (1.00–1.001)	0.48
Median IgG4 (mg/dl)	566	540	0.83		
Median IgE (IU/ml)	288	178	0.34		
Median WBC (/μl)	5060	6200	0.12	1.00 (0.999–1.0002)	0.15
Median RF (U/ml)	1	6	0.11	1.02 (0.99–1.04)	0.23
Median CIC (μg/ml)	10	5	0.55		
Median sIL-2R (U/ml)	1072	1149	1.00		

Among the 65 IgG4-related periaortitis/periarteritis patients, 43 (66.2%) received corticosteroids. All patients showed improvement of wall thickening after therapy, although nine patients also exhibited worsening of luminal dilatation. Univariate analysis comparisons of the nine patients with luminal dilatation after corticosteroid therapy and the 34 patients without revealed prior luminal dilatation before treatment to be associated with this symptom, which was supported by multiple logistic analysis

*OR* odds ratio, *CI* confidence interval, CIC circulating immune complex, *CRP* C-reactive protein, *IgE* immunoglobulin E, *IgG* immunoglobulin G, *IgG4-RD* immunoglobulin G4-related disease, *RF* rheumatoid factor, *sIL-2R* soluble interleukin-2 receptor, *WBC* white blood cells

## Discussion

This study revealed the following key clinical features of IgG4-related periaortitis/periarteritis: the condition predominantly affected the infra-renal artery portion of the abdominal aorta, continuing to the iliac arteries; it was more prevalent in patients with an older IgG4-RD onset age and in those with a highly active disease state as evidenced by elevated serum IgG, IgG4, CIC, and sIL2R; and the main risk factor for worsening luminal dilatation after corticosteroid therapy was prior luminal dilatation. We discuss these findings in the following sections.

### Distribution of IgG4-related periaortitis/periarteritis

IgG4-related periaortitis/periarteritis predominantly affected the infra-renal artery portion of the abdominal aorta continuing to the iliac arteries. Lesions were also sometimes localized at the abdominal aorta with a separate manifestation in the ascending aorta. Inoue et al. [21] reported that among 22 periarterial lesions, 13 were located in the abdominal aorta to the iliac arteries. Mizushima et al. [23] described the affected regions as the abdominal aorta or iliac arteries in most cases. Several explanations are plausible as to why IgG4-related periaortitis/periarteritis predominantly affects the infra-renal artery portion of the abdominal aorta. Unique histological and pathological characteristics may exist between this region and other portions that are differentially influenced by vessel flow or arteriosclerosis, or other vessel changes; AAA also primarily affects the inferior renal artery portion, and it has been proposed that areas of constantly peaked oscillatory shear stress in the infra-renal aorta might be one of the factors leading to morphological changes over time [28]. Moreover, arteriosclerosis progression inferior to the renal artery is common and may influence the occurrence of AAA. Castelein et al. [29] found no significant difference in the distribution of periaortic lesions in their comparison of an IgG4-related periaortitis group and an idiopathic periaortitis group despite the calcium content of the total aortic wall being significantly increased in the former group. They suggested a possible role of atherosclerotic plaque in the pathogenesis of IgG4-related periaortitis. Although not investigated in the present study, a similar mechanism may be at work in IgG4-related periaortitis/periarteritis. Alternatively, vessel lesions may be related to the surrounding adventitia. We observed that kidney/urinary tract involvement was more frequent in IgG4-related periaortitis/periarteritis, the inflammation of which might have influenced disease localization. Further investigation is needed to clarify the distribution, prevalence, and characteristics of IgG4-related periaortitis/periarteritis among the five proposed subtypes.

### Risk factors for periaortitis/periarteritis in IgG4-RD

IgG4-related periaortitis/periarteritis tended to occur in patients having an older IgG4-RD onset age and in those with highly active disease states displaying elevated serum IgG, IgG4, CIC, and sIL2R. In support of this, Mizushima et al. [23] reported that 37 of 40 patients (92.5%) with IgG4-related periaortitis/periarteritis exhibited increased serum IgG4 levels and 31 of 40 patients (77.5%) showed elevated serum IgG. Thus, we consider a high IgG4-RD activity state to most strongly influence the onset of periaortitis/periarteritis. Hypertension and smoking were not risk factors in this study, which suggested that IgG4-related periaortitis/periarteritis was associated with risk factors more similar to those for other IgG4-RDs and less associated with those well known for atherosclerosis.

### Effects of corticosteroid therapy on IgG4-related periaortitis/periarteritis

All patients who received corticosteroids showed improvements in wall thickening after therapy, although a fifth exhibited worsening of luminal dilatation. Some studies have described that the luminal diameters of affected vessels were not changed or dilated [21, 23], while others reported rupture after corticosteroid therapy [30]. This investigation revealed a key risk factor for worsening luminal dilatation after corticosteroid therapy to be prior luminal dilatation, which was concordant with Mizushima et al.’s results [23]. Patients with prior luminal dilatation may be complicated with hypertension or arteriosclerotic factors and therefore prone to aneurysms. Aneurysms may also be associated with advanced age. Deciding whether or not patients with a risk of luminal dilatation exacerbation should receive corticosteroid therapy is challenging. We consider that when patients with prior luminal dilatation need corticosteroids due to serious complications or other vital organ involvement, such as urinary tract stenosis or obstruction, careful monitoring for periaortitis/periarteritis is mandatory.

### IgG4-related periaortitis/periarteritis as a distinct vasculitis disease entity

We observed that the affected regions in IgG4-related periaortitis/periarteritis primarily included large and medium-sized arteries. According to the 2012 Revised International Chapel Hill Consensus Conference Nomenclature of Vasculitides [31], large and medium vessel vasculitis entities consist of Takayasu arteritis and giant cell arteritis, and of polyarteritis nodosa and Kawasaki disease, respectively. Variable vessel vasculitis includes Behcet’s disease and Cogan’s syndrome, while vasculitis associated with probable etiology includes hepatitis B virus-associated vasculitis and Syphilis-associated aortitis. In contrast, IgG4-related periaortitis/periarteritis was characterized by high serum IgG4, predilection for the adventitia and periaortic/periarterial tissue, and more significant thickening of the adventitia that differed remarkably from the clinical findings of established vasculitis entities. Pathologically, IgG4-related periaortic/periarterial changes have been characterized by lymphoplasmacytic infiltration to the adventitia, lymphoid follicle formation, perineural inflammatory extension, obliterative phlebitis, and storiform fibrosis [10, 19, 22], which also show marked differences from other vascular diseases. Taking these into account, IgG4-related periaortitis/periarteritis represents a distinct disease entity in the vasculitis classification system with diagnosis based on elevated activity markers such as IgG4, complicating other IgG4-related vital organ involvement, and exclusion of other systemic diseases and infections.

### Imaging modalities for detecting IgG4-related cardiovascular diseases

Cardiovascular involvement in IgG4-RD may manifest as cardiac pseudotumor, inflammatory periaortitis/periarteritis, coronary arteritis, and/or pericarditis. Because IgG4-related cardiovascular disorders can severely affect patient prognosis, various imaging techniques including echocardiography, CT, ^18^F-FDG-PET, cardiovascular magnetic resonance (CMR), and cardiac catheterization have been successfully adopted for early disease detection and follow-up [9]. Echocardiography and vascular ultrasound are the most commonly used noninvasive, nonradiating imaging techniques for the evaluation of IgG4-related cardiovascular disease. Echocardiography is ideal for the detection of pericardial lesions and cardiac pseudotumors, although its main limitations include an operator-dependent technique, an acoustic window, and inability to perform tissue characterization. As shown in this study, periaortitis/periarteritis can also be assessed by CT as soft tissue thickening around the arteries. The current resolution of CT is most effective in the assessment of the aorta and its proximal branches but is relatively limited for the distal aortic branches. The primary limitations of CT include the need for iodinated contrast administration and radiation exposure. In cases of active periarterial or coronary artery inflammation, ^18^F-FDG-PET reveals FDG uptake at the area of the lesion. Owing to its capability to perform functional and tissue characterization, CMR offers integrated imaging of the aorta, coronary arteries, and heart along with the assessment of disease acuity and extent of fibrosis to guide further treatment. In fact, CMR has been successfully used to determine disease activity in Takayasu arteritis [32]. CMR has the notable drawbacks of a long scanning time, high cost, and contraindication for patients with metallic clips, pacemakers, or other devices [9]. CT was the most suitable modality in the present investigation since we focused on the aorta and its proximal branches.

### Limitations

This study had several limitations. First, because pathologic specimens of vascular lesions were not available to verify IgG4-RD, other conditions causing periaortitis/periarteritis may have been included. Although the improvement of periaortitis/periarteritis after corticosteroid therapy was consistent with IgG4-RD, this too was not definitive. To reduce bias, we identified IgG4-related periaortitis/periarteritis among patients with other confirmed IgG4-RDs, and vascular lesions on CT imaging were reviewed by an expert radiologist using consistent criteria. Second, the sample size of this investigation was limited despite being the largest single-center cohort to date.

## Conclusion

IgG4-related periaortitis/periarteritis predominantly occurred at the infra-renal artery portion of the abdominal aorta, affecting patients with an older IgG4-RD onset age as well as those exhibiting a highly active disease state with elevated serum IgG, IgG4, CIC, and sIL2R. The main risk factor for worsening luminal dilation after corticosteroid therapy was confirmed to be prior luminal dilation.

## Abbreviations

AAA: Abdominal aortic aneurysm
AIP: Autoimmune pancreatitis
CIC: Circulating immune complex
CMR: Cardiovascular magnetic resonance
CRP: C-reactive protein
CT: Computed tomography
^18^F-FDG-PET: Glucose analog 2-deoxy-2-[^18^F] fluoro-d-glucose
IgG4: Immunoglobulin G4
IgG4-RD: Immunoglobulin G4-related disease
MRI: Magnetic resonance imaging
RF: Rheumatoid factor
sIL-2R: Soluble interleukin-2 receptor
WBC: White blood cells
